# Chlorido[hydridotris(pyrazol-1-yl-κ*N* ^2^)borato](1*H*-pyrazole-κ*N* ^2^)(triphenyl­phosphine-κ*P*)ruthenium(II)

**DOI:** 10.1107/S1600536810021525

**Published:** 2010-06-16

**Authors:** Chiung-Cheng Huang, Han-Gung Chen, Yih Hsing Lo, Li-Sheng Hsu, Chia-Her Lin

**Affiliations:** a Department of Chemical Engineering, Tatung University, Taipei 104, Taiwan; b Department of Natural Science, Taipei Municipal University of Education, Taipei 10048, Taiwan; c Department of Chemistry, Chung-Yuan Christian University, Chung-Li 320, Taiwan

## Abstract

In the title compound, [Ru(C_9_H_10_BN_6_)Cl(C_3_H_4_N_2_)(C_18_H_15_P)], the Ru^II^ atom is coordinated by an *N*,*N*′,*N*′′-tridentate hydrido­trispyrazolylborate (Tp) ligand, a pyrazole (HPz) mol­ecule, a chloride ion and a triphenyl­phosphine ligand, resulting in a distorted RuClPN_4_ octa­hedral coordination for the metal ion: the tridentate N atoms occupy one octa­hedral face and the Cl and P atoms are *cis*. One of the phenyl rings is disordered over two orientations in a 0.547 (10):0.453 (10) ratio, and a weak intra­molecular N—H⋯Cl hydrogen bond generates an *S*(5) ring.

## Related literature

For general background to ruthenium coordination chemistry with pyrazole-type ligands, see: Alcock *et al.* (1992 ▶); Cheng *et al.* (2009 ▶); Deacon *et al.* (1998 ▶); Govind *et al.* (1996 ▶); Lo *et al.* (2004 ▶); Pavlik *et al.* (2005 ▶). For related structures, see: Gemel *et al.* (1996 ▶); Slugovc *et al.* (1998 ▶). Tong *et al.* (2008 ▶, 2009 ▶).
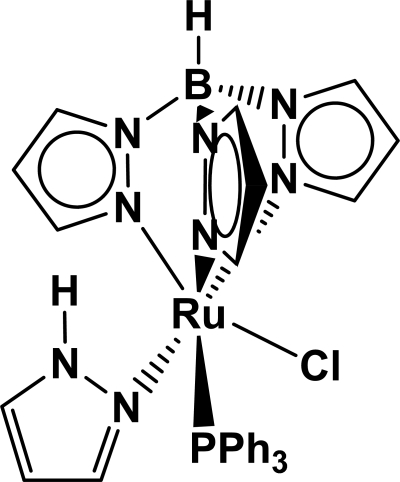


## Experimental

### 

#### Crystal data

[Ru(C_9_H_10_BN_6_)Cl(C_3_H_4_N_2_)(C_18_H_15_P)]
*M* *_r_* = 679.91
Monoclinic, 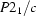

*a* = 17.7782 (12) Å
*b* = 10.0843 (5) Å
*c* = 18.9139 (10) Å
β = 116.316 (3)°
*V* = 3039.5 (3) Å^3^
*Z* = 4
Mo *K*α radiation
μ = 0.69 mm^−1^
*T* = 200 K
0.11 × 0.08 × 0.03 mm

#### Data collection

Nonius KappaCCD diffractometer
Absorption correction: multi-scan (*SORTAV*; Blessing, 1995 ▶) *T*_min_ = 0.928, *T*_max_ = 0.980
22639 measured reflections
5292 independent reflections
3470 reflections with *I* > 2σ(*I*)
*R*_int_ = 0.079

#### Refinement

*R*[*F* ^2^ > 2σ(*F* ^2^)] = 0.053
*wR*(*F* ^2^) = 0.118
*S* = 1.02
5292 reflections
360 parameters
H-atom parameters constrained
Δρ_max_ = 0.89 e Å^−3^
Δρ_min_ = −0.88 e Å^−3^

### 

Data collection: *COLLECT* (Nonius, 1999 ▶); cell refinement: *DENZO* and *SCALEPACK* (Otwinowski & Minor, 1997 ▶); data reduction: *DENZO* and *SCALEPACK*; program(s) used to solve structure: *SHELXS97* (Sheldrick, 2008 ▶); program(s) used to refine structure: *SHELXL97* (Sheldrick, 2008 ▶); molecular graphics: *ORTEP-3* (Farrugia, 1997 ▶); software used to prepare material for publication: *WinGX* (Farrugia, 1999 ▶).

## Supplementary Material

Crystal structure: contains datablocks I, global. DOI: 10.1107/S1600536810021525/hb5462sup1.cif

Structure factors: contains datablocks I. DOI: 10.1107/S1600536810021525/hb5462Isup2.hkl

Additional supplementary materials:  crystallographic information; 3D view; checkCIF report

## Figures and Tables

**Table 1:** Selected bond lengths (Å)

Ru1—N1	2.067 (4)
Ru1—N3	2.097 (4)
Ru1—N5	2.076 (4)
Ru1—N7	2.076 (4)
Ru1—P1	2.3031 (15)
Ru1—Cl1	2.4374 (14)

**Table 2:** Hydrogen-bond geometry (Å, °)

*D*—H⋯*A*	*D*—H	H⋯*A*	*D*⋯*A*	*D*—H⋯*A*
N8—H8′⋯Cl1	0.88	2.49	3.025 (6)	120

**Fig. 1.**
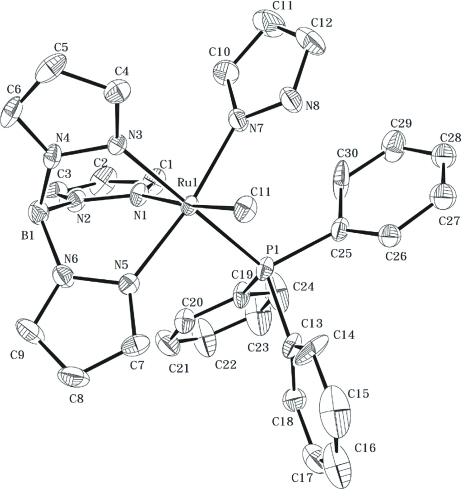
Molecular structure of (I) with displacement ellipsoids drawn at the 30% probability level (H atoms are shown as spheres of arbitrary radius).

## supplementary crystallographic information

### Comment

Pyrazoles and pyrazolate anions are attractive ligands that disclose a rich coordination chemistry (Deacon *et al.*, 1998). Pyrazoles and substituted pyrazoles usually perform as monodentate ligands (Lo *et al.*, 2004) and these monodentate pyrazoles may give rise to fascinating processes such as prototropic equilibrium or reversible metal-ligand binding, which are relevant to biological systems (Govind *et al.*, 1996). On the other hand, Tp (hydridotripyrazolylborate) ligand is often compared with the Cp (Cp = *η*^5^-C_5_H_5_) ligand due to their charge and number of electrons donated in the formation of complex. The ruthenium chloride complex [Ru(Tp)Cl(PPh_3_)_2_] (Alcock *et al.*, 1992) has been used as the precursor for the synthesis of several complexes because of its substitutionally labile phosphines and chloride (Cheng *et al.*, 2009). TpRu complexes are of importance for stoichiometric and catalytic transformations of organic compounds (Pavlik *et al.*, 2005).

Treatment of the complex [Ru(Tp)Cl(PPh_3_)_2_] reacts with pyrazole in toluene affording the title compound [RuCl(Tp)(PPh_3_)(HPz)] (Figure 1). The single crystals of the title compound suitable for X-ray structure analysis were obtained by recrystallization of the crude product from dichloromethane–ether. In the crystal structure of the title compound the ruthenium metal center is coordinated by four N, one P and one Cl atom within slightly distorted octahedron. The bite angle of the Tp ligand produces an average produces an average N—Ru—N angle of 86.6° only slightly distorted from 90°. The three Ru—N(Tp) bond lengths (2.067 (4), 2.097 (4), and 2.076 (4) Å) are slightly longer than the average distance of 2.038 Å in other ruthenium Tp complexes (Gemel *et al.* 1996; Slugovc *et al.* 1998). The Ru—Cl bond of 2.4374 (14) Å are similar to those found in other (pyrazole)ruthenium complexes, such as 2.4259 (14) Å in [Ru(Tp)Cl(PPh_3_)(PhCN)] (Tong *et al.* 2008) and 2.4429 (7) Å in [Ru(Tp)Cl(PPh_3_) (HN=CPh_2_)] (Tong *et al.* 2009). Weak N—H——Cl hydrogen bond is observed in the crystal structure.

### Experimental

To a solution of [Ru(Tp)Cl(PPh_3_)_2_] (3.95 g, 4.50 mmol) in toulene (100 ml), an excess of pyrazole were added. The mixture was heated using a warm water bath for 30 min. A deep yellow color developed during this time. The reaction mixture was stirred for a further 2 h at room temperature (298 K). Then it was concentrated to approximately half of the volume and cooled to 273 K. The yellow precipitate was filtered off, washed with ethanol and ether and dried under vacuum to give the title compound. Yellow prisms of (I) were obtained by recrystallization from dichloromethane–ether.

### Refinement

The H atoms were placed in idealized positions and constrained to ride on their parent atoms, with C—H = 0.95 Å and *U*_iso_(H) = 1.2 *U*_eq_(C) and B—H = 1.0 Å and *U*_iso_(H) = 1.2*U*_eq_(B).

### Crystal data

**Table:**

[Ru(C_9_H_10_BN_6_)Cl(C_3_H_4_N_2_)(C_18_H_15_P)]	*Z* = 4
*M**_r_* = 679.91	*F*(000) = 1384
Monoclinic, *P*2_1_/*c*	*D*_x_ = 1.486 Mg m^−^^3^
Hall symbol: -P 2ybc	Mo *K*α radiation, λ = 0.71073 Å
*a* = 17.7782 (12) Å	µ = 0.69 mm^−^^1^
*b* = 10.0843 (5) Å	*T* = 200 K
*c* = 18.9139 (10) Å	Prism, yellow
β = 116.316 (3)°	0.11 × 0.08 × 0.03 mm
*V* = 3039.5 (3) Å^3^	

### Data collection

**Table:**

Nonius KappaCCD diffractometer	5292 independent reflections
Radiation source: fine-focus sealed tube	3470 reflections with *I* > 2σ(*I*)
graphite	*R*_int_ = 0.079
Detector resolution: 9 pixels mm^-1^	θ_max_ = 25.0°, θ_min_ = 2.4°
CCD rotation images, thick slices scans	*h* = −21→21
Absorption correction: multi-scan (*SORTAV*; Blessing, 1995)	*k* = −11→12
*T*_min_ = 0.928, *T*_max_ = 0.980	*l* = −22→21
22639 measured reflections	

### Refinement

**Table:**

Refinement on *F*^2^	Primary atom site location: structure-invariant direct methods
Least-squares matrix: full	Secondary atom site location: difference Fourier map
*R*[*F*^2^ > 2σ(*F*^2^)] = 0.053	Hydrogen site location: inferred from neighbouring sites
*wR*(*F*^2^) = 0.118	H-atom parameters constrained
*S* = 1.02	*w* = 1/[σ^2^(*F*_o_^2^) + (0.0431*P*)^2^ + 4.7477*P*] where *P* = (*F*_o_^2^ + 2*F*_c_^2^)/3
5292 reflections	(Δ/σ)_max_ = 0.001
360 parameters	Δρ_max_ = 0.89 e Å^−^^3^
0 restraints	Δρ_min_ = −0.88 e Å^−^^3^

### Special details

**Table:**

Geometry. All e.s.d.'s (except the e.s.d. in the dihedral angle between two l.s. planes) are estimated using the full covariance matrix. The cell e.s.d.'s are taken into account individually in the estimation of e.s.d.'s in distances, angles and torsion angles; correlations between e.s.d.'s in cell parameters are only used when they are defined by crystal symmetry. An approximate (isotropic) treatment of cell e.s.d.'s is used for estimating e.s.d.'s involving l.s. planes.
Refinement. Refinement of *F*^2^ against ALL reflections. The weighted *R*-factor *wR* and goodness of fit *S* are based on *F*^2^, conventional *R*-factors *R* are based on *F*, with *F* set to zero for negative *F*^2^. The threshold expression of *F*^2^ > σ(*F*^2^) is used only for calculating *R*-factors(gt) *etc*. and is not relevant to the choice of reflections for refinement. *R*-factors based on *F*^2^ are statistically about twice as large as those based on *F*, and *R*- factors based on ALL data will be even larger.

### Fractional atomic coordinates and isotropic or equivalent isotropic displacement parameters (Å^2^)

**Table:**

	*x*	*y*	*z*	*U*_iso_*/*U*_eq_	Occ. (<1)
C1	0.2902 (4)	0.3286 (6)	0.1960 (4)	0.0463 (16)	
H1	0.2738	0.2987	0.1436	0.056*	
C2	0.2953 (4)	0.4609 (6)	0.2186 (4)	0.0555 (18)	
H2	0.2837	0.5370	0.1858	0.067*	
C3	0.3205 (4)	0.4581 (6)	0.2979 (4)	0.0474 (17)	
H3	0.3290	0.5333	0.3309	0.057*	
C4	0.5121 (3)	0.0343 (6)	0.4007 (4)	0.0404 (15)	
H4	0.5261	−0.0394	0.3776	0.048*	
C5	0.5677 (4)	0.0987 (7)	0.4686 (4)	0.0539 (18)	
H5	0.6255	0.0793	0.4997	0.065*	
C6	0.5218 (4)	0.1956 (6)	0.4810 (4)	0.0480 (17)	
H6	0.5419	0.2566	0.5238	0.058*	
C7	0.2128 (4)	0.0109 (6)	0.3727 (4)	0.0402 (15)	
H7	0.1830	−0.0666	0.3468	0.048*	
C8	0.2055 (4)	0.0742 (7)	0.4345 (4)	0.0562 (19)	
H8	0.1701	0.0498	0.4580	0.067*	
C9	0.2593 (4)	0.1778 (7)	0.4543 (4)	0.057 (2)	
H9	0.2689	0.2395	0.4954	0.069*	
C10	0.4314 (4)	0.1132 (7)	0.1913 (4)	0.0540 (18)	
H10	0.4311	0.2064	0.1983	0.065*	
C11	0.4754 (5)	0.0497 (8)	0.1565 (4)	0.073 (2)	
H11	0.5096	0.0890	0.1354	0.087*	
C12	0.4589 (5)	−0.0808 (8)	0.1592 (4)	0.069 (2)	
H12	0.4799	−0.1519	0.1401	0.082*	
C13	0.1166 (4)	−0.1063 (6)	0.1829 (3)	0.0387 (15)	
C14	0.1434 (4)	−0.2266 (6)	0.2193 (5)	0.068 (2)	
H14	0.2008	−0.2507	0.2383	0.082*	
C15	0.0875 (6)	−0.3145 (8)	0.2288 (5)	0.097 (2)	
H15	0.1071	−0.3971	0.2545	0.116*	
C16	0.0059 (6)	−0.2816 (8)	0.2013 (4)	0.0853 (19)	
H16	−0.0322	−0.3420	0.2069	0.102*	
C17	−0.0222 (5)	−0.1622 (7)	0.1657 (4)	0.0608 (14)	
H17	−0.0796	−0.1390	0.1474	0.073*	
C18	0.0328 (4)	−0.0738 (6)	0.1559 (3)	0.0448 (16)	
H18	0.0126	0.0090	0.1307	0.054*	
C19	0.1261 (3)	0.1512 (5)	0.1297 (3)	0.0377 (15)	
C20	0.1150 (3)	0.2350 (5)	0.1821 (4)	0.0389 (15)	
H20	0.1442	0.2181	0.2371	0.047*	
C21	0.0622 (4)	0.3428 (7)	0.1557 (4)	0.0608 (14)	
H21	0.0536	0.3977	0.1923	0.073*	
C22	0.0221 (6)	0.3709 (8)	0.0766 (5)	0.0853 (19)	
H22	−0.0124	0.4473	0.0582	0.102*	
C23	0.0325 (6)	0.2860 (8)	0.0239 (5)	0.097 (2)	
H23	0.0037	0.3030	−0.0311	0.116*	
C24	0.0842 (5)	0.1779 (7)	0.0509 (4)	0.078 (3)	
H24	0.0909	0.1206	0.0142	0.094*	
C25	0.2000 (3)	−0.0681 (5)	0.0859 (3)	0.0391 (15)	
C26	0.2011 (7)	−0.2090 (10)	0.0842 (7)	0.039 (3)*	0.547 (10)
H26	0.1893	−0.2624	0.1194	0.047*	0.547 (10)
C27	0.2208 (7)	−0.2625 (12)	0.0263 (7)	0.052 (4)*	0.547 (10)
H27	0.2197	−0.3561	0.0204	0.063*	0.547 (10)
C28	0.2412 (8)	−0.1881 (13)	−0.0209 (9)	0.048 (3)*	0.547 (10)
H28	0.2497	−0.2303	−0.0617	0.058*	0.547 (10)
C26'	0.1488 (9)	−0.1639 (13)	0.0340 (8)	0.045 (4)*	0.453 (10)
H26'	0.1061	−0.2001	0.0454	0.054*	0.453 (10)
C27'	0.1535 (10)	−0.2134 (15)	−0.0340 (9)	0.060 (5)*	0.453 (10)
H27'	0.1188	−0.2849	−0.0632	0.072*	0.453 (10)
C28'	0.2087 (10)	−0.1562 (15)	−0.0564 (10)	0.048 (4)*	0.453 (10)
H28'	0.2167	−0.1872	−0.1000	0.057*	0.453 (10)
C29	0.2503 (5)	−0.0547 (7)	−0.0130 (4)	0.063 (2)	
H29	0.2658	−0.0021	−0.0462	0.076*	
C30	0.2354 (6)	−0.0005 (7)	0.0473 (4)	0.075 (3)	
H30	0.2511	0.0891	0.0619	0.090*	
N1	0.3114 (3)	0.2499 (4)	0.2585 (3)	0.0316 (11)	
N2	0.3313 (3)	0.3310 (4)	0.3217 (3)	0.0337 (11)	
N3	0.4364 (3)	0.0895 (4)	0.3725 (2)	0.0281 (10)	
N4	0.4429 (3)	0.1907 (4)	0.4224 (3)	0.0334 (11)	
N5	0.2680 (3)	0.0749 (4)	0.3548 (3)	0.0303 (11)	
N6	0.2968 (3)	0.1791 (4)	0.4062 (3)	0.0389 (12)	
N7	0.3897 (3)	0.0274 (4)	0.2138 (3)	0.0367 (12)	
N8	0.4080 (3)	−0.0912 (5)	0.1936 (3)	0.0476 (14)	
H8'	0.3887	−0.1669	0.2020	0.057*	
B1	0.3644 (4)	0.2722 (6)	0.4052 (4)	0.0397 (18)	
H1'	0.3771	0.3442	0.4452	0.048*	
Cl1	0.34907 (9)	−0.18597 (13)	0.31246 (9)	0.0368 (4)	
Ru1	0.32023 (3)	0.04732 (4)	0.27709 (3)	0.02453 (15)	
P1	0.19260 (9)	0.00460 (14)	0.17152 (9)	0.0329 (4)	

### Atomic displacement parameters (Å^2^)

**Table:**

	*U*^11^	*U*^22^	*U*^33^	*U*^12^	*U*^13^	*U*^23^
C1	0.055 (4)	0.032 (4)	0.040 (4)	−0.002 (3)	0.010 (3)	0.011 (3)
C2	0.067 (5)	0.027 (4)	0.059 (5)	−0.006 (3)	0.016 (4)	0.020 (3)
C3	0.052 (4)	0.020 (3)	0.071 (5)	−0.007 (3)	0.028 (4)	−0.001 (3)
C4	0.034 (3)	0.037 (4)	0.045 (4)	0.003 (3)	0.013 (3)	0.005 (3)
C5	0.032 (4)	0.059 (4)	0.043 (4)	−0.006 (3)	−0.008 (3)	0.014 (4)
C6	0.052 (4)	0.047 (4)	0.026 (4)	−0.023 (4)	0.000 (3)	0.000 (3)
C7	0.032 (3)	0.034 (3)	0.052 (4)	−0.001 (3)	0.016 (3)	0.013 (3)
C8	0.055 (4)	0.063 (5)	0.072 (5)	−0.004 (4)	0.047 (4)	0.002 (4)
C9	0.078 (5)	0.054 (4)	0.068 (5)	0.003 (4)	0.058 (5)	−0.004 (4)
C10	0.064 (5)	0.061 (4)	0.049 (4)	−0.017 (4)	0.037 (4)	−0.001 (4)
C11	0.088 (6)	0.087 (6)	0.074 (6)	−0.008 (5)	0.063 (5)	0.003 (5)
C12	0.078 (6)	0.086 (6)	0.066 (5)	0.010 (5)	0.053 (5)	−0.008 (4)
C13	0.034 (3)	0.033 (3)	0.036 (4)	−0.007 (3)	0.004 (3)	−0.009 (3)
C14	0.049 (4)	0.028 (4)	0.108 (7)	−0.025 (3)	0.017 (4)	0.005 (4)
C15	0.128 (6)	0.072 (4)	0.048 (4)	0.049 (4)	0.001 (4)	0.007 (3)
C16	0.123 (5)	0.068 (4)	0.054 (4)	0.031 (4)	0.030 (4)	−0.003 (3)
C17	0.058 (3)	0.076 (4)	0.051 (3)	0.016 (3)	0.026 (3)	−0.006 (3)
C18	0.043 (4)	0.054 (4)	0.036 (4)	−0.006 (3)	0.016 (3)	−0.002 (3)
C19	0.038 (3)	0.030 (3)	0.028 (4)	0.009 (3)	−0.001 (3)	−0.005 (3)
C20	0.034 (3)	0.036 (3)	0.032 (4)	0.010 (3)	0.002 (3)	0.002 (3)
C21	0.058 (3)	0.076 (4)	0.051 (3)	0.016 (3)	0.026 (3)	−0.006 (3)
C22	0.123 (5)	0.068 (4)	0.054 (4)	0.031 (4)	0.030 (4)	−0.003 (3)
C23	0.128 (6)	0.072 (4)	0.048 (4)	0.049 (4)	0.001 (4)	0.007 (3)
C24	0.101 (6)	0.070 (5)	0.032 (4)	0.056 (5)	0.002 (4)	0.000 (4)
C25	0.030 (3)	0.031 (3)	0.045 (4)	0.001 (3)	0.007 (3)	−0.013 (3)
C29	0.087 (5)	0.061 (5)	0.040 (4)	−0.017 (4)	0.026 (4)	−0.003 (4)
C30	0.151 (8)	0.044 (4)	0.032 (4)	−0.041 (5)	0.043 (5)	−0.017 (3)
N1	0.039 (3)	0.017 (2)	0.033 (3)	−0.002 (2)	0.011 (3)	0.001 (2)
N2	0.038 (3)	0.018 (3)	0.043 (3)	−0.005 (2)	0.017 (3)	−0.003 (2)
N3	0.026 (3)	0.029 (3)	0.024 (3)	−0.001 (2)	0.008 (2)	0.000 (2)
N4	0.041 (3)	0.033 (3)	0.021 (3)	−0.009 (2)	0.008 (2)	−0.003 (2)
N5	0.030 (3)	0.025 (3)	0.034 (3)	0.002 (2)	0.012 (2)	0.001 (2)
N6	0.049 (3)	0.033 (3)	0.044 (3)	−0.001 (2)	0.029 (3)	−0.005 (2)
N7	0.042 (3)	0.035 (3)	0.037 (3)	0.001 (2)	0.021 (3)	−0.006 (2)
N8	0.058 (4)	0.044 (3)	0.052 (4)	0.001 (3)	0.034 (3)	−0.010 (3)
B1	0.049 (5)	0.029 (4)	0.045 (5)	−0.006 (3)	0.025 (4)	−0.008 (3)
Cl1	0.0399 (8)	0.0218 (7)	0.0420 (9)	0.0043 (6)	0.0120 (7)	0.0027 (6)
Ru1	0.0258 (2)	0.0193 (2)	0.0246 (3)	−0.0007 (2)	0.00759 (19)	0.0001 (2)
P1	0.0322 (9)	0.0231 (8)	0.0321 (9)	0.0004 (6)	0.0039 (7)	−0.0036 (6)

### Geometric parameters (Å, °)

**Table:**

C1—N1	1.332 (7)	C20—C21	1.377 (8)
C1—C2	1.392 (8)	C20—H20	0.9500
C1—H1	0.9500	C21—C22	1.371 (9)
C2—C3	1.363 (9)	C21—H21	0.9500
C2—H2	0.9500	C22—C23	1.387 (10)
C3—N2	1.343 (7)	C22—H22	0.9500
C3—H3	0.9500	C23—C24	1.370 (9)
C4—N3	1.329 (6)	C23—H23	0.9500
C4—C5	1.387 (8)	C24—H24	0.9500
C4—H4	0.9500	C25—C30	1.341 (8)
C5—C6	1.359 (9)	C25—C26'	1.390 (14)
C5—H5	0.9500	C25—C26	1.422 (11)
C6—N4	1.350 (7)	C25—P1	1.835 (6)
C6—H6	0.9500	C26—C27	1.396 (14)
C7—N5	1.337 (6)	C26—H26	0.9500
C7—C8	1.387 (8)	C27—C28	1.334 (15)
C7—H7	0.9500	C27—H27	0.9500
C8—C9	1.352 (9)	C28—C29	1.355 (14)
C8—H8	0.9500	C28—H28	0.9500
C9—N6	1.346 (7)	C26'—C27'	1.416 (18)
C9—H9	0.9500	C26'—H26'	0.9500
C10—N7	1.326 (7)	C27'—C28'	1.358 (19)
C10—C11	1.382 (9)	C27'—H27'	0.9500
C10—H10	0.9500	C28'—C29	1.316 (16)
C11—C12	1.354 (9)	C28'—H28'	0.9500
C11—H11	0.9500	C29—C30	1.392 (9)
C12—N8	1.332 (7)	C29—H29	0.9500
C12—H12	0.9500	C30—H30	0.9500
C13—C14	1.371 (8)	N1—N2	1.359 (6)
C13—C18	1.384 (8)	N2—B1	1.539 (8)
C13—P1	1.838 (6)	N3—N4	1.361 (6)
C14—C15	1.402 (11)	N4—B1	1.524 (8)
C14—H14	0.9500	N5—N6	1.367 (6)
C15—C16	1.347 (11)	N6—B1	1.531 (8)
C15—H15	0.9500	N7—N8	1.339 (6)
C16—C17	1.362 (9)	N8—H8'	0.8800
C16—H16	0.9500	B1—H1'	1.0000
C17—C18	1.394 (8)	Ru1—N1	2.067 (4)
C17—H17	0.9500	Ru1—N3	2.097 (4)
C18—H18	0.9500	Ru1—N5	2.076 (4)
C19—C24	1.365 (8)	Ru1—N7	2.076 (4)
C19—C20	1.382 (7)	Ru1—P1	2.3031 (15)
C19—P1	1.839 (5)	Ru1—Cl1	2.4374 (14)
			
N1—C1—C2	110.2 (6)	C27—C26—C25	114.5 (9)
N1—C1—H1	124.9	C27—C26—H26	122.8
C2—C1—H1	124.9	C25—C26—H26	122.8
C3—C2—C1	105.2 (5)	C28—C27—C26	123.0 (12)
C3—C2—H2	127.4	C28—C27—H27	118.5
C1—C2—H2	127.4	C26—C27—H27	118.5
N2—C3—C2	108.5 (5)	C27—C28—C29	122.6 (12)
N2—C3—H3	125.8	C27—C28—H28	118.7
C2—C3—H3	125.8	C29—C28—H28	118.7
N3—C4—C5	110.8 (6)	C25—C26'—C27'	127.1 (12)
N3—C4—H4	124.6	C25—C26'—H26'	116.5
C5—C4—H4	124.6	C27'—C26'—H26'	116.5
C6—C5—C4	105.1 (6)	C28'—C27'—C26'	118.4 (15)
C6—C5—H5	127.5	C28'—C27'—H27'	120.8
C4—C5—H5	127.5	C26'—C27'—H27'	120.8
N4—C6—C5	108.5 (5)	C29—C28'—C27'	114.9 (13)
N4—C6—H6	125.8	C29—C28'—H28'	122.6
C5—C6—H6	125.8	C27'—C28'—H28'	122.6
N5—C7—C8	110.1 (5)	C28'—C29—C28	32.0 (7)
N5—C7—H7	124.9	C28'—C29—C30	123.9 (9)
C8—C7—H7	124.9	C28—C29—C30	115.0 (8)
C9—C8—C7	105.7 (5)	C28'—C29—H29	105.7
C9—C8—H8	127.1	C28—C29—H29	122.5
C7—C8—H8	127.1	C30—C29—H29	122.5
N6—C9—C8	108.8 (6)	C25—C30—C29	123.5 (6)
N6—C9—H9	125.6	C25—C30—H30	118.2
C8—C9—H9	125.6	C29—C30—H30	118.2
N7—C10—C11	111.4 (6)	C1—N1—N2	106.4 (4)
N7—C10—H10	124.3	C1—N1—Ru1	135.2 (4)
C11—C10—H10	124.3	N2—N1—Ru1	118.4 (3)
C12—C11—C10	104.8 (6)	C3—N2—N1	109.7 (5)
C12—C11—H11	127.6	C3—N2—B1	130.1 (5)
C10—C11—H11	127.6	N1—N2—B1	120.1 (4)
N8—C12—C11	107.4 (6)	C4—N3—N4	106.0 (5)
N8—C12—H12	126.3	C4—N3—Ru1	134.1 (4)
C11—C12—H12	126.3	N4—N3—Ru1	119.8 (3)
C14—C13—C18	118.2 (6)	C6—N4—N3	109.6 (5)
C14—C13—P1	119.2 (5)	C6—N4—B1	132.5 (5)
C18—C13—P1	122.7 (5)	N3—N4—B1	117.9 (5)
C13—C14—C15	121.0 (7)	C7—N5—N6	106.1 (4)
C13—C14—H14	119.5	C7—N5—Ru1	136.3 (4)
C15—C14—H14	119.5	N6—N5—Ru1	117.5 (3)
C16—C15—C14	119.9 (8)	C9—N6—N5	109.3 (5)
C16—C15—H15	120.0	C9—N6—B1	129.9 (5)
C14—C15—H15	120.0	N5—N6—B1	120.7 (4)
C15—C16—C17	120.3 (9)	C10—N7—N8	104.5 (5)
C15—C16—H16	119.9	C10—N7—Ru1	133.0 (4)
C17—C16—H16	119.9	N8—N7—Ru1	122.2 (4)
C16—C17—C18	120.4 (7)	C12—N8—N7	111.9 (5)
C16—C17—H17	119.8	C12—N8—H8'	124.0
C18—C17—H17	119.8	N7—N8—H8'	124.0
C13—C18—C17	120.2 (6)	N4—B1—N6	108.4 (5)
C13—C18—H18	119.9	N4—B1—N2	108.9 (5)
C17—C18—H18	119.9	N6—B1—N2	107.7 (5)
C24—C19—C20	118.7 (5)	N4—B1—H1'	110.6
C24—C19—P1	124.3 (5)	N6—B1—H1'	110.6
C20—C19—P1	116.9 (4)	N2—B1—H1'	110.6
C21—C20—C19	120.9 (6)	N1—Ru1—N5	87.90 (16)
C21—C20—H20	119.5	N1—Ru1—N7	91.02 (17)
C19—C20—H20	119.5	N5—Ru1—N7	171.23 (18)
C22—C21—C20	119.9 (6)	N1—Ru1—N3	85.26 (17)
C22—C21—H21	120.0	N5—Ru1—N3	86.71 (17)
C20—C21—H21	120.0	N7—Ru1—N3	84.53 (17)
C21—C22—C23	119.1 (7)	N1—Ru1—P1	93.79 (13)
C21—C22—H22	120.4	N5—Ru1—P1	93.51 (13)
C23—C22—H22	120.4	N7—Ru1—P1	95.25 (13)
C24—C23—C22	120.3 (7)	N3—Ru1—P1	179.02 (12)
C24—C23—H23	119.8	N1—Ru1—Cl1	172.51 (13)
C22—C23—H23	119.8	N5—Ru1—Cl1	92.33 (12)
C19—C24—C23	120.9 (6)	N7—Ru1—Cl1	87.62 (13)
C19—C24—H24	119.5	N3—Ru1—Cl1	87.28 (12)
C23—C24—H24	119.5	P1—Ru1—Cl1	93.67 (5)
C30—C25—C26'	106.7 (7)	C25—P1—C13	101.8 (3)
C30—C25—C26	118.8 (7)	C25—P1—C19	103.0 (3)
C26'—C25—C26	45.6 (6)	C13—P1—C19	100.0 (3)
C30—C25—P1	120.8 (4)	C25—P1—Ru1	114.28 (18)
C26'—C25—P1	128.3 (7)	C13—P1—Ru1	120.32 (19)
C26—C25—P1	115.2 (6)	C19—P1—Ru1	114.91 (19)

### Hydrogen-bond geometry (Å, °)

**Table:**

*D*—H···*A*	*D*—H	H···*A*	*D*···*A*	*D*—H···*A*
N8—H8'···Cl1	0.88	2.49	3.025 (6)	120
